# Protocol for a systematic review of venous coupler devices versus hand-sewn anastomosis for microsurgical free flap reconstruction

**DOI:** 10.1186/s13643-018-0871-x

**Published:** 2018-11-13

**Authors:** Timo Rodi, Alexander Geierlehner, Afshin Mosahebi, Grigorios Tanos, Justin Conrad Rosen Wormald

**Affiliations:** 1 UCL Division of Surgery and Interventional Science, Royal Free Hospital, 9th Floor, Pond Street, London, NW3 2QG UK; 2 Department of Plastic and Reconstructive Surgery, Royal Free Hospital, London, UK; 3 Department of Plastic Surgery, Stoke Mandeville Hospital, Aylesbury, Buckinghamshire UK; 4 Nuffield Department of Orthopaedics, Rheumatology and Musculoskeletal Sciences, University of Oxford, Oxford, UK

**Keywords:** Free flap, Free tissue transfer, Microsurgery, Venous coupler device, Venous anastomosis, Systematic review

## Abstract

**Background:**

A patent microvascular anastomosis is of paramount importance in free tissue transfer. Anastomotic coupler devices provide an alternative to technically demanding hand-sewn venous anastomosis. Various advantages of these devices have been discussed but previous systematic reviews had methodological flaws or did not perform a meta-analysis. This review aims to evaluate the quality of the evidence and quantify the efficacy and safety of venous couplers compared to hand-sewn anastomosis.

**Methods:**

A PRISMA-compliant systematic review and meta-analysis will be performed. A comprehensive search strategy has been developed and will be applied to the databases MEDLINE and Embase from inception to October 2018. All clinical studies using anastomotic coupler devices for venous anastomoses in free tissue transfer will be eligible for inclusion. Screening of studies and data extraction will be performed independently by two authors. Our primary outcome is anastomotic venous thrombosis. Secondary outcomes will include time to complete the venous anastomosis, tearing of veins, anastomotic leakage, flap loss/failure and fiscal outcomes. The risk of bias for included studies will be assessed by using the ROBINS-I tool, and recommendations based on the evidence will be made using the GRADE approach. Descriptive statistical analyses will be used and if two or more studies report the same outcome, data will be pooled for comparative analysis. A direct comparison meta-analysis will be performed if possible.

**Discussion:**

There has been no comparison of coupled and hand-sewn venous anastomoses using a robust and validated methodology preceded by a protocol and performing meta-analysis. Included studies are expected to be mainly observational and prone to bias; however, there is value in summarising the evidence, assessing its risk of bias and performing meta-analysis to guide clinicians. By using a broad approach including all types of flaps, we foresee inherent differences regarding the unit of analysis and different anatomic sites. This will limit the validity of our conclusions but is unavoidable. We will seek unpublished data from authors and perform subgroup analysis where appropriate. Limitations and areas of uncertainty will be discussed to guide future research.

**Systematic review registration:**

PROSPERO CRD42018110111

## Background

Free tissue transfer is a widely used procedure in reconstructive surgery and provides a way to replace like with like tissue from distant donor sites. The most critical part and key principle of this procedure are the microvascular anastomoses between the flap and recipient site vessels. Since the first sutured microvascular anastomosis was described in 1960 [1], new devices have been developed in order to simplify this complex procedure. Mechanical coupler devices have gained popularity over the past decades and are now routinely used for performing venous anastomosis in free flap surgery [2–5]. Most venous coupler devices use polyethylene rings with interlocking steel pins to securely connect blood vessels [6].

Various advantages of venous couplers have been described in the literature, including ease of handling, improved intimal alignment and reduced foreign material within the vessel lumen [6]. There have been three systematic reviews evaluating the evidence base supporting the use of venous couplers compared to hand-sewn anastomosis [7–9]; however, all these reviews had methodological issues or did not perform a meta-analysis. Even though Zhu et al. performed a meta-analysis in their recent review, it was not preceded by a protocol and did not include an assessment of bias or grading of the evidence [9].

The aim of this review is to systematically evaluate the efficacy and safety of venous coupler devices compared to hand-sewn anastomosis, appraise the quality of evidence in the relevant literature and perform meta-analysis of key outcomes to guide further surgical practice.

## Methods

This protocol will be registered on the PROSPERO International Prospective Register of Systematic Reviews [10] and will adhere to the guidelines as described in the PRISMA-P (Preferred Reporting Items for Systematic review and Meta-Analysis Protocols) checklist [11]. The methodology of this review will be according to the Cochrane Handbook for Systematic Review of Interventions [12]. The final review will be performed and reported with respect to the Preferred Reporting Items for Systematic Reviews and Meta-Analyses (PRISMA) statement and checklist [13].

### Search strategy

A comprehensive search strategy will be developed in collaboration with a research librarian and will aim to capture all relevant articles relating to the review question. Both MeSH term and free-text search strategies will be developed using the keywords described in Table 1, combined using Boolean operators. The search strategy will be applied to the MEDLINE and Embase databases with the time period from database inception to 1 October 2018. In addition, a search of ClinicalTrials.gov will be performed to make sure that no relevant studies were missed.

**Table 1:** Electronic database search—Ovid EMBASE and MEDLINE

	Concept 1	AND	Concept 2	AND	Concept 3
MeSH term					
OR	free flap, microsurgical		veins		surgical anastomosis
Key words					
OR	“flap*”		“vein”		“couple*”
OR	“free flap*”		“venous”		“anastomotic coupler”
OR	“free tissue transfer”				“venous coupler”
OR					“coupled device”

Once the MeSH and text term searches have been combined, duplicates will be removed. The combined results will be screened independently by two authors (TR and AG) who will confer when their literature search has been completed. Consensus will be sought, and all remaining articles will be read in full before a decision on inclusion is made. Disagreements between the screening authors will be moderated by a third author (JW), and a final decision will be made. The bibliography of the final included studies will be screened to check for additional publications missed by the search strategy. Publications in any language from any country will be eligible for inclusion. The list of screened and included studies will be managed in a bespoke, pre-defined Excel sheet (Microsoft, Redmond, WA, USA). Citations will be managed in EndNote (Clarivate Analytics, Boston, MA, USA).

### Study selection criteria

All clinical studies using anastomotic coupler devices for microvascular anastomoses in free flap tissue transfer will be eligible for inclusion. Studies using couplers for both arterial and venous anastomoses will only be included if the data for venous anastomosis is clearly distinguishable. Studies directly comparing venous coupler devices and hand-sewn anastomoses will be included and directly compared through meta-analysis if possible. Study selection criteria were defined with reference to the Population, Intervention, Comparison, Outcome (PICO) Model for Clinical Questions [14].

#### Participants

Participants will be patients undergoing free flap tissue transfer. There will be no restrictions for inclusion based on the body region of the procedure, the clinical setting or patient demographics.

#### Intervention

All publications reporting on the use of anastomotic couplers for venous anastomoses in human free tissue transfer will be included. In vitro, animal or cadaveric studies will not be included. Any studies reporting on multiple anastomosis procedures such as hand-sewn anastomosis or venous couplers for arterial anastomosis will only be included where the data for venous anastomosis is clearly distinguishable from the other procedures.

#### Comparator

All studies comparing the use of anastomotic coupler devices for venous anastomosis in free flap tissue transfer to hand-sewn venous anastomosis will be eligible for inclusion in the final review.

#### Outcome

There will be no limitations for studies with regard to the reported outcome measures or time of follow-up. All experimental studies reporting on relevant clinical outcomes will be included. Any unpublished or ongoing trials will be excluded.

#### Study design

Experimental studies (randomised controlled trials (RCTs)) and observational studies (cohort, case-control and case series) will be eligible for inclusion. No limitation regarding patient inclusion criteria or study size will be made. Case reports, letters, opinion pieces and literature reviews will be excluded.

### Data extraction

Our data collection and analysis process will be based on the Cochrane Handbook of Systematic Reviews of Interventions [12]. Data will be extracted and documented in a predesignated electronic form by two authors individually (TR and AG). Consensus will be sought through discussion and might involve a third team member (JW) if necessary to resolve any deviations or disagreement between authors.

The following data will be compiled for comparison:

1. Study characteristics and patient demographics
2. Type of intervention/free tissue transfer subsites
3. Anastomosis technique and characteristics
4. Intra- and postoperative outcomes

Where authors provide data for venous and arterial anastomosis, we will only extract data for the venous anastomosis if these data are clearly distinguishable. Venous anastomosis will be the preferred unit of analysis. If we are not able to directly extract data for a particular outcome, then the authors of the study will be contacted by email. If there is no response, a second email will be sent at that point. If there is no response following the reminder email, then the study will be excluded, and the outcome recorded in the PRISMA flow chart.

### Outcome measures

Our primary outcome will be the postoperative venous thrombosis rate. On-table anastomotic thrombosis during surgery will be considered the same as any later latent thrombosis and discussed in detail where applicable. Secondary outcome parameters will be time to complete the venous anastomosis intraoperatively, tearing of veins, anastomotic leakage requiring revision and flap loss/failure and fiscal outcomes.

### Risk of bias assessment

The risk of bias for each included study will be assessed by using the ROBINS-I tool [15]. We have used the PICO framework to develop preconceptions of bias based on what we expect to find in the literature. A clinical study with low risk of bias would fulfil the following criteria (organised using the PICO framework):

- Participants: consecutive, no selection bias, similar baseline demographics
- Intervention and comparison: same defined intervention throughout (similar flaps, use of hand-sewn or coupled anastomosis in one individual patient, preference/experience of operating surgeon), no selection bias
- Outcomes: no loss of data, all data presented, confounders accounted for (e.g. size mismatch of anastomosis vessels), methods of outcome assessment comparable across intervention groups (e.g. similar and appropriate follow-up time), evidence that results correspond to intended outcomes (e.g. pre-registered protocol)

Where studies do not display these criteria, they will be judged at moderate or high risk of bias (NB the above list is not exhaustive). Where there is too little data to make a judgement, authors will be contacted to provide additional unpublished data where appropriate. We will appraise the quality of evidence for each outcome based on the Grading of Recommendations, Assessment, Development and Evaluation (GRADE) approach [16].

### Data analysis and synthesis

We will perform simple descriptive statistical analyses for patient demographics. The rate of microvascular venous thrombosis will be calculated for each group (venous coupler versus hand-sewn anastomosis) so that the results can be compared across the included studies. The primary outcome, venous thrombosis, will be calculated and displayed as a rate (%), and if two or more studies report the same outcome, then the data from the single studies will be pooled for comparative analysis. We will perform a direct comparison meta-analysis, using RevMan5 to calculate the relative risk ratios with 95% confidence intervals using the Cochran-Mantel-Haenszel test. We will use a random-effects model due to the anticipated study heterogeneity, and subgroup analyses will be undertaken where appropriate. Statistical heterogeneity will be quantified for all direct comparisons using the *I*-squared statistic, and significance will be set at the 5% level. The meta-analysis results, if possible, will be displayed in forest plots with a funnel plot to assess publication bias in our primary outcome [17].

## Discussion

Despite the growing popularity of venous coupler devices and their routine use in free flap tissue transfer [2–5], there have only been three systematic reviews evaluating their use [7, 8, 18]. All these reviews had methodological flaws or did not perform meta-analysis. Moreover, included data was not clearly distinguishable between arterial and venous anastomoses. We hope to identify the superior technique in a direct comparison and therefore provide clinicians with information to choose the appropriate surgical technique. Our conclusions will be based on a robust and validated methodology including a quality of evidence appraisal for each included study.

It is important to discuss the perceived quality of the literature. A majority of studies that were included in previous reviews on the topic were observational (cohort) rather than experimental (trials) [7–9]. We therefore anticipate a similar quality of the literature for our included studies, so there will be bias. However, there is still value in summarising the current evidence, assessing its risk of bias and performing direct comparison meta-analysis to help guide clinicians [19]. Another difficulty with free flap research is the interpretation of the unit of analysis. Some studies might discuss outcomes per flap rather than per patient. Multiple flaps in a patient cannot be regarded as independent and therefore should not be combined into a single group for comparative analysis. We will discuss these issues in our review and will seek unpublished data from authors if we encounter it [20].

By choosing a broad, pragmatic approach to include all flaps, we accept that there will be inherent differences between different anatomical sites like the lower extremity, breast, head and neck. We will perform subgroup analysis if possible and will discuss any apparent differences in the descriptive analysis where applicable. There will be confounding factors and inherent bias due to the likely non-experimental, observational nature of the primary data that will be included. This will limit the validity of our conclusions and is unavoidable. We will however perform a thorough risk of bias assessment using the ROBINS-I tool to appropriately weight the reliability of the data we encounter during our analysis. We will scrutinise the data for presence of unmeasured variables that may affect the primary and secondary outcomes and will discuss their impact where applicable.

## Abbreviations

GRADE: Grading of Recommendations, Assessment, Development and Evaluation
MeSH: Medical Subject Headings
NIH: National Heart Lung and Blood Institute
NIHR: National Institute for Health Research
PICO: Population, Intervention, Comparison, Outcome
PRISMA: Preferred Reporting Items for Systematic Reviews and Meta-Analyses
PROSPERO: International prospective register of systematic reviews
RCT: Randomised control trial
